# Supplementary material for: COZOID: contact zone identifier for visual analysis of protein-protein interactions
Source: BMC Bioinformatics. 2018 Apr 6;19:125. doi: 10.1186/s12859-018-2113-6 (PMC5889581; doi:10.1186/s12859-018-2113-6)
Supplement: Supplementary file 2 — Software build. Executable binary file of the software tool. (ZIP 118,784 kb) [file 12859_2018_2113_MOESM2_ESM.zip › cozoid/platform/docs/testng-6.5.1-javadoc.zip!/org/testng/annotations/Factory.html]

Factory (TestNG)


---


|  |  |  |
| --- | --- | --- |
| **Overview** \| **Package** \| **Class** \| **Tree** \| **Deprecated** \| **Index** \| **Help** | |  |
| **PREV CLASS**   **NEXT CLASS** | **FRAMES**    **NO FRAMES**     **All Classes** |  |
| SUMMARY: REQUIRED \| OPTIONAL | DETAIL: ELEMENT |  |


---


## org.testng.annotations Annotation Type Factory

---

``` @Retention(value=RUNTIME) @Target(value={METHOD,CONSTRUCTOR}) public @interface Factory ```

Marks a method as a factory that returns objects that will be used by TestNG
as Test classes. The method must return Object[].

**Author:**
:   Cedric Beust

---

| **Optional Element Summary** | |
| --- | --- |
| `java.lang.String` | `dataProvider`             The name of the data provider for this test method. |
| `java.lang.Class<?>` | `dataProviderClass`             The class where to look for the data provider. |
| `boolean` | `enabled`             Whether this factory is enabled. |
| `java.lang.String[]` | `parameters`             **Deprecated.** *Use @Parameters* |

### parameters

```
@Deprecated
public abstract java.lang.String[] parameters
```

:   **Deprecated.** *Use @Parameters*

    :   The list of variables used to fill the parameters of this method.
        These variables must be defined in the property file.

        :   **Default:**: {}

---


### dataProvider

```
public abstract java.lang.String dataProvider
```

:   The name of the data provider for this test method.

    :   **See Also:**: `DataProvider`

        **Default:**: ""

---


### dataProviderClass

```
public abstract java.lang.Class<?> dataProviderClass
```

:   The class where to look for the data provider. If not
    specified, the dataprovider will be looked on the class
    of the current test method or one of its super classes.
    If this attribute is specified, the data provider method
    needs to be static on the specified class.

    :   **Default:**: java.lang.Object.class

---


### enabled

```
public abstract boolean enabled
```

:   Whether this factory is enabled.

    :   **Default:**: true


---


|  |  |  |
| --- | --- | --- |
| **Overview** \| **Package** \| **Class** \| **Tree** \| **Deprecated** \| **Index** \| **Help** | |  |
| **PREV CLASS**   **NEXT CLASS** | **FRAMES**    **NO FRAMES**     **All Classes** |  |
| SUMMARY: REQUIRED \| OPTIONAL | DETAIL: ELEMENT |  |


---
